# A Learning-Based Approach for Biomedical Word Sense Disambiguation

**DOI:** 10.1100/2012/949247

**Published:** 2012-05-01

**Authors:** Hisham Al-Mubaid, Sandeep Gungu

**Affiliations:** University of Houston-Clear Lake, Houston, TX 77058, USA

## Abstract

In the biomedical domain, word sense ambiguity is a widely spread problem with bioinformatics research effort devoted to it being not commensurate and allowing for more development. This paper presents and evaluates a learning-based approach for sense disambiguation within the biomedical domain. The main limitation with supervised methods is the need for a corpus of manually disambiguated instances of the ambiguous words. However, the advances in automatic text annotation and tagging techniques with the help of the plethora of knowledge sources like ontologies and text literature in the biomedical domain will help lessen this limitation. The proposed method utilizes the interaction model (mutual information) between the context words and the senses of the target word to induce reliable learning models for sense disambiguation. The method has been evaluated with the benchmark dataset NLM-WSD with various settings and in biomedical entity species disambiguation. The evaluation results showed that the approach is very competitive and outperforms recently reported results of other published techniques.

## 1. Introduction

Word sense disambiguation is the task of determining the correct sense of a given word in a given context. In the general language domain, and within natural language processing (NLP), the word sense disambiguation (WSD) problem has been studied and investigated extensively over the past few decades [1, 2]. In the biomedical domain, on the other hand, WSD is more widely spread in the biological and medical texts and sometimes with more severe consequences. The amount of WSD research in the biomedical domain is not proportional to the extent of the problem. As an example, in the biomedical texts, the term “*blood pressure*” has three possible senses according to the Unified Medical Language System (UMLS) [3] as follows: *organism function*, *diagnostic procedure*, and *laboratory or test result.* Thus, if this term *blood pressure *is found in a medical text, the reader has to manually judge and determines which one of these three senses is intended in that text. Word sense disambiguation contributes in many important applications including the text mining, information extraction, and information retrieval systems [1, 2, 4]. It is also considered a key component in most intelligent knowledge discovery and text mining applications.

The main classes of approaches of word sense disambiguation include supervised methods and unsupervised methods. The supervised methods rely on training and learning phases that require a dataset or corpus containing manually disambiguated instances to be used to train the system [5, 6]. The unsupervised methods, on the other hand, are based on knowledge sources like ontology, for example, from UMLS, or text corpora [2, 4, 7, 8]. Our approach in this paper is a supervised approach. In this paper, we present and evaluate a supervised method for biomedical word sense disambiguation. The method is based on machine learning and uses some feature selection techniques in constructing feature vectors for the words to be disambiguated. We conducted the evaluation using the NLM-WSD benchmark corpus and species disambiguation dataset. The evaluation results proved the competitiveness of the proposed approach as it outperforms some recently published techniques including supervised techniques.

## 2. Related Work

In the biomedical domain, the applications of text mining and machine learning techniques were quite successful and encouraging [6]. Most of the methods for biomedical entity name recognition, classification, or disambiguation can be roughly divided into three categories: (i) supervised and machine-learning-based techniques, (ii) statistical and corpus-based techniques, and (iii) syntactic and rule-based techniques [9–11]. Moreover, the bioinformatics literature shows that biomedical WSD has been a quite active area of research with a number of approaches proposed and applied to biomedical data [1, 2, 4, 8, 12, 13].

Agirre et al. proposed a graph-based WSD technique which is considered unsupervised but relies on UMLS [2]. The concepts of UMLS are represented as a graph, and WSD is done using personalized page rank algorithm [2].

In another related research, Jimeno-Yepes and Aronson [4] presented a review and evaluation of four WSD approaches that rely on UMLS as the source for knowledge for disambiguation. In [1], Stevenson et al. use supervised learners with linguistic features extracted from the context of the word in combination with MeSH terms for disambiguation. 

The UMLS has been used, by Humphrey et al., as a knowledge source for assigning the correct sense for a given word [13]. They used journal descriptor indexing of the abstract containing the term to assign a semantic type from UMLS metathesaurus [3, 13].

In bioinformatics and computational biology, there are quite a few tasks similar to WSD like biomedical term disambiguation, gene protein name disambiguation, and disambiguating species for biomedical named entities [9–11]. The task of biomedical named entity disambiguation or classification is an augmentation of the well-known task of biomedical named entity recognition (NER). In NER, biomedical entity names, for example, gene names, are recognized and extracted from the text. In the biomedical named entity disambiguation, the extracted entity names (e.g., gene product names) will be applied onto a process such that each occurrence should be disambiguated as either *gene *name or *protein* name as the same name can refer to a gene or protein. For example, the biomedical entity name *SBP2* can be a *gene* name or a *protein* name depending on the context [10, 11]. Furthermore, in species disambiguation, the term *c-myc* is a gene, but it can be either in a human gene (*homo sapiens*) or mouse gene (*mus musculus*) depending on the context [9–11, 14–16].

In [9], Wang et al. devised a rule based system to disambiguate biomedical entity names, like gene products, based on species. In that approach [9], some parsing techniques are used and syntactic parse tree with paths between words to determine if there exists a path between species word and the entity name. They employed and examined several parsers in the task including *C&C*, *Enju*, *Minipar*, and *Stanford-Genia* [9, 15, 16]. 

## 3. A Method for WSD

A word sense disambiguation method is an algorithm that assigns the most accurate sense to a given word in a given context. Our method is a supervised method requiring a training corpus that contains manually disambiguated instances of the ambiguous words. The method is based on a word classification and disambiguation technique that we have proposed in a preliminary work [17]. In the previous work, [17], we introduced a method for term disambiguation and evaluated it with biomedical terms to disambiguate *gene* and *protein* names in medical texts.

The method relies on representing the instances of the word to be disambiguated, *w*_*x*_, as a *feature vector,* and the components of this vector are neighborhood context words in the training instances. In the context of the target word, *w*_*x*_, we select the words with the high *discriminating* capabilities as the components of the vectors. As a supervised technique, this method consists of two stages *learning* (or *training*) stage and a *testing* (or *application*) stage. The trained models (*classifiers*) produced from the learning phase will then be used to disambiguate unseen and unlabeled examples in the testing phase. That is, during the learning phase, the constructed feature vectors of the training instances will be used as labeled examples to train classifiers. The classifier will be then used to disambiguate unseen and unlabeled examples in the application phase. One of the main strength of this method is that the features are selected for learning and classification.

**Feature Selection**

The features selected from the training examples have great impact on the effectiveness of the machine learning technique. Extensive research efforts have been devoted to feature selection in machine learning research [18–21]. The labeled training instances will be used to extract the word features for the feature vectors.

Suppose the word *w*_*x*_ has two senses *s*_1_, *s*_2_, let the set *C*_1_ be the set of *w*_*x*_ instances labeled with *s*_1_, and suppose *C*_2_ contains instances of *w*_*x*_ labeled with sense *s*_2_. So, each instance of *w*_*x*_ labeled with sense *s*_1_ or *s*_2_ (i.e., in the set *C*_1_ or in the set *C*_2_) can be viewed as

(1) $$p_{n}\cdots p_{3}p_{2}p_{1}<w_{x};\;\;s_{i}\;>f_{1}f_{2}f_{3}\cdots f_{n},$$

where the words *p*_1_, *p*_2_,…, *p*_*n*_ and *f*_1_, *f*_2_,…, *f*_*n*_ are the context words surrounding this instance, and *n* is the *window size*. Next, we collect all the context words *p*_*i*_ and *f*_*i*_ of all instances in *C*_1_ and *C*_2_ in one set *W *(*s.t. W* = {*w*_1_, *w*_2_,…, *w*_*m*_}). Each context word *w*_*i*_ *∈W* may occur in the contexts of instances labeled with *s*_1_ or with *s*_2_ or combination and in any distribution. We want to determine that, if we see a context word *w*_*q*_ in an ambiguous instance/example, to what extent this occurrence of *w*_*q*_ suggests that this example belongs to *C*_1_ or to *C*_2_. Thus, we use as features those context words *w*_*i*_ that can highly discriminate between *C*_1_ and *C*_2_. For that, we use feature selection techniques such as *mutual information* (MI) [19, 20] as follows. For each context word *w*_*i*_ *∈ W* in the labeled training examples, we compute four values *a*, *b*, *c*, and *d* as follows:

a = number of occurrences of *w*_*i*_ in *C*_1_,
b = number of occurrences of *w*_*i*_ in *C*_2_,
c = number of examples of *C*_1_ that do not contain *w*_*i*_,
d = number of examples of *C*_2_ that do not contain *w*_*i*_.

Therefore, the *mutual information *(MI) can be defined as

(2) $$\text{MI}=\frac{N*a}{\left(a+b\right)*\left(a+c\right)},$$

and *N* is the total number of training examples. MI is a well-known concept in information theory and statistical learning. MI is a measure of interaction and common information between two variables [22]. In this work, we adapted MI to represent the interaction between the context words *w*_*i*_ and the class label based on the values *a* through *d* as defined above. We utilized the training corpus of the labeled instances of the word to be disambiguated to compile the list of all context words (*W* = {*w*_1_, *w*_2_,…, *w*_*m*_}) as explained above; all instances of one sense are under one class label. We notice that if the context word, *w*_*i*_, is mostly occurring in class *C*_1_ (or mostly in *C*_2_), then the MI indicates this as shown in (2). Thus, MI can be used as a means to estimate the amount of information interaction between a context work and a class label. So, MI is used to select the context words with the highest discriminating capability between *C*_1_ and *C*_2_. For simplicity, and without loss of generality, we assume that we have two senses (two class labels). Moreover, following the same intuitive reasoning of mutual information, MI, we define another method, M2, for selecting the words as features to be included in the feature vectors as follows:

(3) $$\text{M}2=\frac{a+d\;}{b+c}.$$

In the following example, assume that the target word *w*_*x*_ has 10 instances already labeled with one of two senses as shown in Table 1. Class *C*_1_ are the instances of *w*_*x*_ with the first sense, while *C*_2_ are the instances of *w*_*x*_ instances in the second sense. Each instance is shown with its context words within certain window size. The target word *w*_*x*_ is shown in bold face. In this example, *N* = 10 is the total number of training examples. The values of *a*, *b*, *c*, *d* for *w*_*p*_ are (4,1,1,4), respectively. That is, *w*_*p*_ has 4 occurrences in *C*_1_ and one instance in *C*_2_, and so on. The values of *a*, *b*, *c*, *d* for *w*_*q*_ are (3, 2, 2, 3), respectively. As we can see, *w*_*p*_ is more highly related with the class *C*_1_ than *w*_*q*_, and so it has more discriminating power than *w*_*q*_, and this is quantified by their MI values. MI values for *w*_*p*_ and *w*_*q*_ are 1.8 and 1.2, respectively.

Then, MI (or M2) value is computed for all context words *w*_*i*_ *∈ W*. Then, the context words *w*_*i*_ are ordered based on their MI values, and the top *k* words *w*_*i*_ with highest MI values are selected as features. In this research, we experimented with *k* values of 100, 200, and 300. With *k* = 100, for example, each training example will be represented by a vector of 100 entries such that the first entry represent the context word *w*_*i*_ with the highest MI value, and the second entry represents the context word with the second highest MI value and so on.

Then, for a given training example, the feature vector entry is set to +MI (or −MI) if the corresponding feature (*context word*) occurs (does not occur) in that training example and set to −MI otherwise. Table 2 shows the top 10 context words with the ten highest MI values for the ambiguous word “*cold*” in the NLM-WSD benchmark corpus explained in Section 3. These 10 words will be used to compose the feature vectors for training or testing examples of the terms to be disambiguated. For example, a simple feature vector of size 5 can be as follows:

(4) $$\left[\begin{array}{ccccc} 1.23 & -1.21 & 0.95 & 0.92 & -0.88 \end{array}\right].$$

This feature vector represents an instance that has the first, third, and fourth context words available in its context, and 1.23 is the MI value of the context word with the highest MI.

**The Learning Phase**

From the labeled training examples of the word, we build the feature vectors using the top context words selected by MI or M2 as features. After that, we use the support vector machine (SVM) [23] as the learner to train the classifier using the training vectors. SVM has been shown as one of the most successful and efficient machine learning algorithms and is well founded theoretically and experimentally [7, 17, 18, 23]. The applications of SVM are abound; in particular, in NLP domain like text categorization, relation extraction, named entity recognition, SVM proved to be the best performer. We use *SVM-light* (http://svmlight.joachims.org/) implementation with the default parameters and with the Radial Basis Function (RBF) kernel.

**The Disambiguation Step**

In the testing step, we want to disambiguate an instance *w*_*q*_ of the word *w*. We construct a feature vector *V*_*q*_ for the instance *w*_*q*_ the same way as in the learning step. The induced learning model (classifier) from the learning step will be employed to classify it (assign *w*_*q*_) to one of the two senses.

## 4. Evaluation and Experiments

### 4.1. Biomedical WSD (NLM-WSD)

**Dataset**

We used the benchmark dataset NLM-WSD for biomedical word sense disambiguation [24]. This dataset was created as a unified and benchmark set of ambiguous medical terms that have been reviewed and disambiguated by reviewers from the field. Most of the previous work on biomedical WSD uses this dataset [1, 2, 4]. The NLM-WSD corpus contains 50 ambiguous terms with 100 instances for each term for a total of 5000 examples. Each example is basically a *Medline* abstract containing one or more occurrences of the ambiguous word. The instances of these ambiguous terms were disambiguated by 11 annotators who assigned a sense for each instance [24]. The assigned senses are semantic types from UMLS. When the annotators did not assign any sense for an instance, then that instance is tagged with “*none*”. Only one term “*association*” with all of its 100 instances were annotated *none* and so dropped from the testing.

**Text Preprocessing**

On this benchmark corpus, we have carried out some text preprocessing steps.

- Converting all words to *lowercase*.
- Removing *stopwords*: removing all common function words like “*is*” “*the*” “*in*”,… and so forth.
- Performing word *stemming* using *Porter *stemming algorithm [25].

Moreover, unlike other previous work, words with less than 3 or more than 50 characters are not ignored currently (unless dropped by the stopword removal step). Also words with parentheses or square brackets are not ignored and part of speech is not used.

After the text preprocessing is completed, for each word we convert the instances into numeric feature vectors. Then, we use SVM for training and testing with 5-fold cross validation 5FCV such that 80% of the instances are used for training and the remaining 20% are used for testing, and this is repeated five times by changing the training-testing portions of the data. The accuracy is taken as the mean accuracy of the five folds and the accuracy is computed as

(5) $$\text{Accuracy}=\frac{\text{no}.\;\text{of}\;\text{instances}\;\text{with}\;\text{correct}\;\text{assigned}\;\text{senses}}{\text{total}\;\text{no}.\;\text{of}\;\text{tested}\;\text{instances}}.$$

We also use the *baseline* method which is the most frequent sense (mfs) for each word.

**Experiments**

Initially, we evaluated our WSD method with all the 49 words (excluding *association* as mentioned previously) such that, a word is included in the evaluation only if it has at least two or more senses with each sense having at least two instances annotated with it. This lead, to a total of 31 words tested in this evaluation, and 18 words were dropped because they do not have at least two instances annotated for each one of two senses. For example, the word “*depression*” has two senses: *mental or behavioral dysfunction* and *functional concept*. Out of the 100 instances of *depression, *85 instances are tagged with the first sense, and remaining 15 instances are tagged with “*None*” (i.e., no instances tagged with a second sense), and so it was excluded in this evaluation. Likewise, the word “*discharge*” was not tested as it has only one instance tagged with the first sense, 74 instances tagged with the second sense, and 25 instances tagged with *None*. We used *k* = 200, and the *window* size is 5. The accuracy results of this first evaluation (EV1) are shown in Table 4. The detailed results of this evaluation are included in Table 5.

In the second evaluation (EV2) and third evaluation (EV3), we changed the parameter and the word/features selection formula. In EV2, we set *k* = 300, and window size is still 5. In EV3, we kept *k* = 300, window = 5, and changed the word/feature selection formula to M2 defined in (3). Table 5 contains the results of EV2 and EV3. To judge on performance of our method and compare our results with similar techniques, we included several reported results from three recent publications from 2008 to 2010 [1, 2, 4] with our results in Table 6 under the same experimental settings.

### 4.2. Species Disambiguation

In biomedical text, named entities, like gene name, are used the same way irrespective of the species of the entity. As a result, it will be difficult to extract relevant medical information automatically from texts using information extraction system. In biomedical named entity species disambiguation, for a given entity name, for example, *c-myc*, we want to disambiguate this entity name, *c-myc*, based on the species (e.g., *human* versus *mouse*) [9]. In one instance, *c-myc* might refer to a human gene, while in another instance it refers to a mouse gene.

For example, in Table 3, the biomedical entity name BCL-2 (*a protein name*) in the first text (no. 1) is human while in the second one is a mouse protein. We examined our system on this task of species disambiguation. We obtained the data from the project of Wang et al. [9]. From their data, we tested the biomedical entity names that occur in at least two species with at least 3 occurrences in each species. This enables us to use two instances for training and one for testing and repeat it three times. If the entity has 5 or more occurrences in one species, we repeat five times using 5FCV as in Section 4.1. We extracted and tested our system on a total 465 instances of entity names with an average of 8 instances per species for each entity name. In the original dataset (gold standard), 90% of the terms have all their instances occurring in only one species [9] and so cannot be tested in our system. Our system requires that each term should have instances in two or more species with at least 3 occurrences in each species. The results of Wang et al. are shown in Table 7, whereas the results of our proposed system are shown in Table 8 in terms of precision, recall, and F1. 

## 5. Discussion and Conclusion

The main weakness of the supervised and machine-learning-based methods for WSD is their dependency on the annotated training text which includes manually disambiguated instances of the ambiguous word [2, 17]. However, over the time, the increasing volumes of text and literature in very high rates and the new algorithms and techniques for text annotation and concept mapping will alleviate this problem. Moreover, the advances in ontology development and integration in the biomedical domain will facilitate even more the process of automatic text annotation.

In this paper, we reported a machine learning approach for biomedical WSD. The approach was evaluated with a benchmark dataset, NLM-WSD, to facilitate the comparison with the results of previous work. The average accuracy results of our method, compared to some recent reported results (Table 6), are promising and proving that our method outperforms those recently reported methods. Table 6 contains the results for 11 methods: baseline method (mfs), our method (last column), and 9 other methods from recent work published in 2008 to 2010 (from [1, 2, 4]). The average accuracy of our method is the highest (90.3%), and the closest one is NB (86.0%).

Our method also outperforms all 10 other methods in 12 out of 31 words followed by NB which outperforms the rest in 7 words.

Stevenson et al. in their paper [1] report extensive accuracy results of their method (we call it *Stevenson-2008*) along with four other methods including Joshi-2005 and McInnes-2007, with various combinations of words from *NLM-WSD *corpus used for testing. For example, Joshi-2005 tested their system on 28 words (out of the whole set 50 words) and other techniques used 22 words, 15 words, or the whole set [1]. In Table 6, the results of the three methods (Joshi-2005, McInnes-2007, and Stevenson-2008) are taken from Stevenson et al. [1]. These three methods are supervised methods and used various machine learning algorithm and wide sets of features. For example, Stevenson-2008 used linguistic features, CUI's, MeSH terms, and combination of these features. They employed three learners VSM (vector space model), Naïve Bayes (NB), and SVM. The results included in Table 6 are their best results with VSM and (linguistic + MeSH) features [1]. The method of Joshi-2005 uses five supervised learning methods and collocation features, while McInnes-2007 uses NB [1].

Our evaluation is done on 31 words (*as explained in Section 3*). We obtained the results of the other methods on these 31 words from the references shown in Table 6 to allow for direct comparison. The best result reported in their paper is 87.8% using all words with VSM model and for McInnes 85.3% also with the whole set [1]. The best result of Stevensons-2008 for subsets was 85.1% using a subset of 22 words defined by Stevenson et al. [1].

The results of the three methods (single, subset, full) in Table 6 are taken directly from Agirre et al. [2]. As shown in Table 6, the average accuracy of these three methods (68.8%, 59.7%, and 63.5%) on the 31 words is significantly lower than our method (90.3%) and also the average accuracy of their method on the whole set (65.9%, 63.0%, and 65.9%); we note that their method is unsupervised and does not require tagged instances [2]. In another work, Jimeno-Yepes and Aronson evaluate four unsupervised methods on the whole NLM-WSD set [4] as well as NB and combination of the four methods. The accuracy of the four methods ranges from 58.3% to 88.3% (NB) on the whole set, and NB was found to be the best performer followed by *CombSW* (76.3%) [4]. The average accuracy results of NB and two combinations (NB, CombSW, and CombV) on our 31 word-subset are 86%, 73.1%, and 72.1% respectively which are lower than our results, see Table 6.

When we applied our system onto the species disambiguation task, the results are also encouraging as shown in Table 8. The evaluation results of our method compare very well with those reported in [9] as shown in Table 7. From their results (Table 7), we notice that the best overall performance was obtained with the ML method (machine learning) with *precision*, *recall*, and *F1* values being equal at 82.69. Our results as shown in Table 8 are not directly comparable with those in Table 7 due to the difference in the size of test set. However, we can see that our method's performance is reasonably well standing in terms of precision, recall, and F1. The main strength of this method is in using MI values as weights encoded in the feature vectors. These weights enable the learner to induce quite reliable models for sense disambiguation. As the components of the vectors, +MI and −MI, are the common information between context word and class labels, the induced learners are finely calibrated towards the disambiguation task.

All the results showed that the technique is fairly successful and effective in the disambiguation task. Thus, more research work should be exerted to carry out further improvements on the performance of this technique. In future work of this research, we plan to investigate the possibility of disambiguating entity names when all instances of that entity are occurring in one species. Currently, our method is supervised and required annotated instances in both classes to be able to test new samples.

## Figures and Tables

**Table 1:** An example of a training corpus of 10 instances of an ambiguous word *w*_*x*_ where 5 instances are in the first sense listed under class label *C*_1_ and 5 instances of the second sense listed under class *C*_2_. The context word *w*_*p*_ has 4 and 1 occurrences in Class *C*_1_ and *C*_2_, respectively, while *w*_*q*_ has 3 and 2 occurrences in *C*_1_ and *C*_2_, respectively.

*C*_1_	*C*_2_
⋯*w*_*p*_ ⋯ **w**_**x**_ ⋯ *w*_*q*_⋯	⋯*w*_*p*_ ⋯ **w**_**x**_⋯
⋯*w*_*p*_ ⋯ **w**_**x**_ ⋯ *w*_*q*_⋯	⋯*w*_*q*_ ⋯ **w**_**x**_⋯
⋯*w*_*p*_ ⋯ **w**_**x**_⋯	⋯*w*_*q*_ ⋯ **w**_**x**_⋯
⋯*w*_*p*_ ⋯ **w**_**x**_⋯	⋯**w**_**x**_⋯
⋯*w*_*q*_ ⋯ **w**_**x**_⋯	⋯**w**_**x**_⋯

**Table 2:** Context words with the top MI values for the ambiguous word “*cold*”.

Context words *w*_*i*_	
Import	
Understand	
Ischemia	
Reperfus	
Respons	
Stor	
Arteri	
Attempt	
Repress	
Quantit	

**Table 3:** A sample text from species disambiguation.

Homosapiens (human)	Mus Musculus (mouse)
(No. 1) Significantly, Diva lacks critical residues in the conserved BH3 region that mediate the interaction between BH3-containing proapoptotic Bcl-2 homologues and their prosurvival binding partners. Consistent with this, Diva did not bind to cellular Bcl-2 family members including Bcl-2, Bcl-XL, Bcl-w, Mcl-1, and A1/Bfl-1	(No. 2) The BCL-2 family has various pairs of antagonist and agonist proteins that regulate apoptosis. Whether their function is interdependent is uncertain. Using a genetic approach to address this question, we utilized gain- and loss-of-function models of Bcl-2 and Bax and found that apoptosis and thymic hypoplasia characteristic of Bcl-2-deficient mice are largely absent in mice also deficient in Bax

**Table 4:** Accuracy results of the first evaluation, EV1, where each sense has to have at least two instances tagged with it.

	Accuracy
Fold 1	0.912
Fold 2	0.931
Fold 3	0.917
Fold 4	0.897
Fold 5	0.862

**Average**	**0.903**

**Table 5:** Detailed accuracy results of three evaluations EV1, EV2, and EV3.

Word	Baseline (mfs)	EV1	EV2	EV3
Adjustment	0.67	0.99	0.96	0.93
Blood_Pressure	0.54	0.98	0.80	0.83
Cold	0.91	0.94	0.92	0.95
Condition	0.98	0.95	0.95	0.95
Culture	0.89	0.87	0.96	0.94
Degree	0.97	0.93	0.93	0.93
Evaluation	0.50	0.98	0.82	0.85
Extraction	0.94	0.94	0.93	0.94
Failure	0.86	0.83	0.83	0.83
Fat	0.97	0.93	0.93	0.93
Ganglion	0.93	0.93	0.91	0.93
Glucose	0.91	0.90	0.90	0.93
Growth	0.63	0.92	1.00	0.96
Immune Suppression	0.59	0.98	0.88	0.87
Implantation	0.83	0.91	0.96	0.87
Japanese	0.92	0.92	0.97	0.92
Lead	0.93	0.84	0.84	0.84
Man	0.63	0.98	0.90	0.92
Mosaic	0.54	0.99	0.77	0.87
Nutrition	0.51	0.94	0.70	0.88
Pathology	0.86	0.79	0.96	0.92
Radiation	0.62	0.83	0.93	0.89
Reduction	0.82	0.63	0.63	0.63
Repair	0.76	0.92	0.91	0.96
Sex	0.80	0.94	0.97	0.88
Support	0.80	0.67	0.67	0.67
Surgery	0.98	0.95	0.95	0.95
Ultrasound	0.84	0.93	0.93	0.91
Variation	0.80	0.86	0.94	0.89
Weight	0.55	0.83	0.57	0.85
White	0.54	1.00	0.69	0.77

**Mean Accuracy**	**0.775**	**0.903**	**0.87**	**0.88**

**Table 6:** Comparison of our results with the best reported results from recent reported techniques.

Word	Baseline (mfs)	Previous Results									Our method (EV1)
		Stevenson et al. [1]			Agirre et al. [2]			Jimeno-Yepes and Aronson [4]			
		Joshi-2005	McInnes 2007	Stevenson-2008	Single	Subset	Full	NB	CombSW	CombV	
Adjustment	67	71	70	74		33.3	35.5	76.3	69	53.9	99
Blood pressure	54	53	46	46	53.0	50	48	57.0	38	44	98
Cold	91	90	89	88	32.6	26.3	28.4	92.6	39	79	94
Condition	98	—	89	89	95.7	39.1	48.9	97.8	78	69	95
Culture	89	—	94	95		33	77	93.0	100	54	87
Degree	97	89	79	95		95.4	93.8	96.9	88	82	93
Evaluation	50	69	73	81	59	54	50	78.0	52	50	98
Extraction	94	84	86	85		23	27.6	94.3	98	86	94
Failure	86	—	73	67		27.6	72.4	86.2	86	100	83
Fat	97	84	77	84	56.2	63	95.9	97.3	91	84	93
Ganglion	93	—	94	96	66	77	64	95.0	88	86	93
Glucose	91	—	90	91	91	91	90	91.0	78	39	90
Growth	63	71	69	68	37	37	37	73.0	55	66	92
Immune suppression	59	80	75	80	64	59	62	79.0	60	65	98
Implantation	83	94	92	93	75	84.7	84.7	98.0	94	97	91
Japanese	92	77	76	75	70.9	70.9	64.6	92.4	63	94	92
Lead	93	89	90	94	93.1	93.1	93.1	93.1	83	86	84
Man	63	89	80	90	61.5	34.8	44.6	87.0	65	42	98
Mosaic	54	87	75	87		60.8	66	82.5	84	72	99
Nutrition	51	52	49	54		33.7	32.6	55.1	45	43	94
Pathology	86	85	84	85		34.3	28.3	85.9	76	83	79
Radiation	62	82	81	84	58.2	53.1	53.1	83.7	76	76	82
Reduction	82	91	92	89	36.4	54.5	54.5	81.8	100	82	63
Repair	76	87	93	88	63.2	72.1	76.5	95.6	87	88	92
Sex	80	88	87	87	84	85	85	84.0	60	53	94
Support	80	—	91	89	80	80	80	80.0	100	90	67
Surgery	98	—	94	97	95.9	97	97	98.0	43	96	95
Ultrasound	84	92	85	90	84	84	83	85.0	81	83	93
Variation	80	—	91	95	85	80	75	91.0	65	86	86
Weight	55	83	79	81	56.6	56.6	56.6	84.9	66	68	83
White	54	79	74	76	68.9	67.8	63.3	81.1	57	58	100

**Average**	**77.5**	**81.1**	**81.2**	**83.6**	**68.8**	**59.7**	**63.5**	**86.0**	**73.1**	**72.7**	**90.3**

**Table 7:** The averaged evaluation results from Wang et al. [9].

	Micro-avg.			Macro-avg.		
	Precision	Recall	F1	Precision	Recall	F1
RULE-MAJORITY	72.2	62.39	66.94	27.77	46.67	29.32
RULE-SP	74.09	64.03	68.69	29.77	53.81	32.2
RULE-SPSENT	72.94	63.03	67.63	30.22	54.76	32.93
C&C	73.82	63.79	68.44	30.51	53.59	33.43
ENJU	72.98	63.06	67.66	31.35	55	34.61
ENJU-Genia	73	63.08	67.68	30.11	53.42	32.97
Minipar	73.02	63.1	67.69	30.19	53.56	33.1
Stanford	73.67	63.66	68.3	31.17	56.35	34.35
Stanford-Genia	73.48	63.5	68.13	30.61	55.61	33.78
ML	82.69	82.69	82.69	27.01	27.84	27.37
RELATION	75.24	63.99	69.16	31.97	55.61	34.8
HYBRID	83.8	83.8	83.8	57.56	49.72	49.9

**Table 8:** Precision, recall, and F1 results of our method on the fivefold in the species disambiguation experiments.

	Micro-avg		
	Precision	Recall	F1
Fold 1	81.86	92.78	87.0
Fold 2	82.08	94.77	88.0
Fold 3	82.95	97.31	89.6
Fold 4	84.12	98.70	90.8
Fold 5	81.25	85.83	83.5

Average	82.45	93.88	87.8

*Total instances tested: 465*			
